# Seasonality of influenza and other respiratory viruses

**DOI:** 10.15252/emmm.202115352

**Published:** 2022-02-14

**Authors:** Gabriele Neumann, Yoshihiro Kawaoka

**Affiliations:** ^1^ Influenza Research Institute University of Wisconsin‐Madison Madison WI USA; ^2^ Institute of Medical Science University of Tokyo Tokyo Japan; ^3^ Research Center for Global Viral Diseases National Center for Global Health and Medicine Japan

**Keywords:** Evolution & Ecology, Microbiology, Virology & Host Pathogen Interaction

## Abstract

In virology, the term seasonality describes variations in virus prevalence at more or less regular intervals throughout the year. Specifically, it has long been recognized that outbreaks of human influenza viruses, respiratory syncytial virus (RSV), and human coronaviruses occur in temperate climates during the winter season, whereas low activity is detected during the summer months. Other human respiratory viruses, such as parainfluenza viruses, human metapneumoviruses, and rhinoviruses, show highest activity during the spring or fall season in temperate regions, depending on the virus and subtype. In tropical climates, influenza viruses circulate throughout the year and no distinct seasonal patterns are observed, although virus outbreaks tend to spike during the rainy season. Overall, seasonality is more pronounced with greater distance from the equator, and tends to be less pronounced in regions closer to the equator (Li *et al*, 2019).

## Graphical abstract

Respiratory viruses may follow different patterns of dissemination during pandemics and epidemics. Climate conditions have less effect on pandemic virus spread, which appears to depend more on human behavior. Once pandemic viruses become endemic, they may adopt seasonal patterns that are primarily driven by climate changes throughout the year.

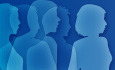

The fairly regular seasonal patterns of epidemics, caused by respiratory viruses circulating in humans, are in contrast to sporadically occurring pandemics, which are caused by viruses to which most humans lack immunity. The 1918 H1N1 influenza pandemic killed an estimated 50 million people worldwide during three major waves in the spring and fall of 1918, and in the winter/spring of 1919. The H2N2 influenza pandemic of 1957 emerged in East Asia in the spring of 1957, was first detected in the US in the summer of 1957, and caused peaks of excess death in October 1957 and February 1958. In 1968, a novel H3N2 pandemic influenza virus was first reported in the US in September 1968 and caused excess mortality in the winter of 1968/1969. In the spring of 2009, a novel H1N1 influenza virus emerged and spread widely during the summer and fall of 2009. Similarly, the novel severe acute respiratory syndrome coronavirus 2 (SARS‐CoV‐2) was first reported in late 2019/early 2020, caused its first pandemic wave in Europe in the spring of 2020, and has since caused several waves with high case numbers around the globe. Thus, epidemic human respiratory viruses and novel pandemic respiratory viruses follow different patterns of dissemination that may be driven by different factors.

In recent years, at least three mechanisms have been discussed to explain the seasonality of epidemic respiratory viruses in humans (reviewed in Moriyama *et al*, (2020)): (i) virus stability and transmissibility under changing environmental conditions (most prominently, humidity and temperature); (ii) human behavior (including indoor/outdoor activities, indoor crowding, number of close contacts, holiday, and vacation travel, etc.); and (iii) the impact of changing environmental conditions on host defense mechanisms. Currently, the relative contribution of these factors to the seasonality of respiratory viruses is not fully understood.

Virus stability and transmissibility under changing environmental conditions have long been discussed as an important factor in the seasonality of epidemic human respiratory viruses. Human‐to‐human transmission of respiratory viruses occurs through aerosols or contact with virus‐contaminated objects. The water content of (virus‐laden) aerosols shrinks through evaporation after the aerosols have been exhaled into the ambient air; this evaporation process is affected by the ambient temperature and relative humidity (reviewed in GAeF (2020)). The final size of the virus‐laden particles will determine how long they stay in the air: While larger aerosols sink to the ground rapidly (i.e., within seconds‐to‐minutes), smaller aerosols remain in the air for several hours and can disperse over greater distances than larger aerosols (reviewed in GAeF (2020)); the infectivity of virus‐laden small aerosols may differ among the respiratory viruses.

Several experimental studies have assessed the stability and transmissibility of respiratory viruses at different temperatures and relative humidity. In general, viruses that cause seasonal spikes during the winter months in temperate climates are more stable and transmissible in animal models at low temperature (about 5°C) and humidity (approx. 10–40% relative humidity) (i.e., conditions that are typical for the winter season in temperate regions) than at the higher temperature (about 25°C) and relative humidity (≥ 60%) found during the summer season in temperate climates.

For influenza viruses, *in vitro* studies and studies in guinea pigs and ferrets have revealed a biphasic pattern with high virus stability at low relative humidity of 10–40% (typical for the winter season in temperate climates), low virus stability and viability at intermediate relative humidity of 40–60% (typical for the spring and fall seasons in temperate climates), and high virus stability and viability at high relative humidity of 60–100% (typical of tropical climates). Influenza virus transmissibility in animal models is higher at 5°C compared to 20°C (Lowen *et al*, 2007); at the latter temperature, influenza virus transmission occurs at low relative humidity, but not at high relative humidity (Lowen *et al*, 2007). At 30°C, influenza virus does not transmit among guinea pigs via respiratory droplets, but is efficiently transmitted via direct contact transmission to naïve animals housed in the same cage (Lowen *et al*, 2007; Lowen & Palese, 2009). On the basis of these findings, Lowen and Palese (2009) hypothesized that influenza virus transmission occurs through aerosol transmission during the winter season in temperate climates, but may be driven by direct contact transmission in tropical regions.

Overall, in temperate (but not in tropical) climates, absolute humidity may be an important factor in the seasonality of epidemic influenza viruses, because spikes of influenza activity are often associated with low absolute humidity in the weeks prior to increased influenza activity (Shaman *et al*, 2011), and a model based on absolute humidity recapitulated the seasonality of epidemic influenza activity in temperate regions (Shaman *et al*, 2011). In contrast, pandemic influenza viruses have spread widely during the spring and summer in temperate regions, when the climate conditions (i.e., intermediate ranges of humidity and temperature) may not be ideal for virus transmission. Therefore, climate conditions do not seem to be a major factor in the dissemination of pandemic influenza viruses that encounter a naïve population.

SARS‐CoV caused an outbreak in Hong Kong in the spring of 2013. This virus was relatively stable at room temperature (22–25°C) and intermediate humidity (40–50%), but high temperature (38°C) and high humidity (> 95%) led to a rapid decline in viability. Moreover, two studies found that the optimal conditions for human‐to‐human transmission of SARS‐CoV were 16–28°C and intermediate humidity of about 50%. Collectively, these findings suggest that certain subtropical climates (such as the spring season in Hong Kong) and the conditions in air‐conditioned rooms may favor the spread of SARS‐CoV. Numerous studies have now assessed the impact of climate on the transmission and mortality of the current pandemic SARS‐CoV‐2 virus (a meta‐analysis is described in Ref. (Romero Starke *et al*, 2021)). Although the data are inconsistent, the authors of the meta‐analysis concluded that low temperature and relative humidity are associated with increased SARS‐CoV‐2 transmission (Romero Starke *et al*, 2021). However, as stated earlier, climate may not be a major factor in the spread of pandemic viruses that encounter a naïve population.

The effect of human behavior on the seasonality of epidemic human respiratory infections is a topic of ongoing studies and discussions, in part because of its complexity with multiple confounding factors. The lower temperatures during the winter season in temperate climates may confine people to indoor activities with indoor crowding, which could facilitate virus transmission. In fact, during cold or rainy weather, people tend to spend more time indoors compared with warmer, drier weather. However, in industrialized countries in the temperate climate zone, people annually spend more than 85% of their time indoors (Klepeis *et al*, 2001; Schweizer *et al*, 2007), suggesting that most person‐to‐person contacts occur indoors. The number of person‐to‐person contacts indoors may vary among the seasons, due to holidays and holiday travel (several major holidays including Thanksgiving [in the US], Christmas, Hanukkah, New Year, and Chinese New Year fall in the winter season in the northern hemisphere) and the start of the academic school year. In general, the number of person‐to‐person contacts is greater on workdays than on weekends, and greater during regular weeks compared to holidays, suggesting that most human‐to‐human transmission events may occur during regular work weeks. While the number of person‐to‐person contacts may be lower on holidays compared to workdays, travel and social gatherings during holidays and vacation lead to person‐to‐person contacts outside the regular circle of contacts, thereby introducing the virus into new communities. Thus, for epidemic human respiratory viruses, human behavior may exacerbate climate‐driven seasonality in temperate regions, at least in the northern hemisphere where low humidity, low temperature, and major holidays coincide.

The pandemic SARS‐CoV‐2 virus is encountering a naïve population and virus ‘seeding’ into communities during gatherings outside regular circles of contact may be a major contributor to virus dissemination. This may explain spikes in SARS‐CoV‐2 cases after the Thanksgiving and Christmas Holidays in the US. For pandemic viruses, human behavior is likely a more important factor for virus dissemination than climate. Once SARS‐CoV‐2 becomes an epidemic human respiratory virus like the other four human coronaviruses, seasonal outbreaks may depend more on the climate and less on human behavior.

In addition to the time spent indoors and the number of indoor contacts, the indoor environment may contribute to the seasonality of respiratory viruses. The relative indoor humidity in buildings during the winter season is typically low. As stated earlier, low humidity at room temperature facilitates influenza virus transmission in animal models, whereas no transmission occurs at room temperature and high relative humidity (Lowen *et al*, 2007). Thus, the low air humidity in enclosed rooms in winter likely facilitates human‐to‐human influenza virus transmission, and therefore the seasonality of influenza virus outbreaks.

In recent years, the effect of environmental conditions on host antiviral defense mechanisms has gained more attention (reviewed in Moriyama *et al*, (2020)). The mucus lining the epithelial cells in the upper airways provides a first barrier by trapping viruses before they infect cells. Dry air slows the flow of mucus along the cilia of the epithelial cell layer, resulting in delayed virus clearance. Moreover, dry air causes the loss of cilia on airway epithelial cells and the detachment of epithelial cells. Thus, lower humidity weakens the first line of defense against infecting viruses.

Innate immune responses are an important host defense mechanism after a virus infects a cell. Several studies have demonstrated seasonal molecular changes in human immune responses with differences in cytokine levels and the transcriptome between the seasons. Higher levels of interleukin 6 (IL‐6) are associated with the increased mortality of highly pathogenic influenza and SARS‐CoV‐2 infections; of note, IL‐6 signaling in humans is higher in winter than in summer. Moreover, studies with rhinoviruses have shown that innate immune responses are lower at 33°C (i.e., the temperature of the upper respiratory tract) compared to 37°C (i.e., the temperature of the lower respiratory tract), allowing efficient rhinovirus replication in the upper respiratory tract. Importantly, low relative humidity dampens innate immune responses, particularly the induction of IFN‐stimulated genes upon virus infection (Kudo *et al*, 2019). However, one study reported similar innate immune responses in guinea pigs housed at either 5°C or 20°C (Lowen *et al*, 2007). Additional studies are needed to full assess the impact of different environmental conditions on host innate immune responses.

Collectively, the data indicate that low humidity and temperature are important drivers in the seasonality of epidemic human respiratory viruses because these climate conditions increase virus stability and transmissibility, create an indoor environment conducive to virus transmission, and dampen host cell immune responses. Increasing the air humidity in enclosed rooms has been suggested as an easily implementable measure to reduce the transmission of respiratory viruses in wintertime. In contrast, the fulminant spread of pandemic respiratory viruses may be primarily driven by human behavior; once these viruses become epidemic, their continuing circulation in humans may adopt a seasonal pattern that correlates with climate changes in temperate regions.

## Disclosure statement and competing interests

The authors do not have any competing interests.
